# Transformer-based molecular optimization beyond matched molecular pairs

**DOI:** 10.1186/s13321-022-00599-3

**Published:** 2022-03-28

**Authors:** Jiazhen He, Eva Nittinger, Christian Tyrchan, Werngard Czechtizky, Atanas Patronov, Esben Jannik Bjerrum, Ola Engkvist

**Affiliations:** 1 Molecular AI, Discovery Sciences, R&D, AstraZeneca, Gothenburg, Sweden; 2 Medicinal Chemistry, Research and Early Development, Respiratory and Immunology (R&I), BioPharmaceuticals R&D, AstraZeneca, Gothenburg, Sweden; 3 Department of Computer Science and Engineering, Chalmers University of Technology, Gothenburg, Sweden

**Keywords:** Molecular optimization, Matched molecular pairs, Transformer, Tanimoto similarity, Scaffold, ADMET

## Abstract

Molecular optimization aims to improve the drug profile of a starting molecule. It is a fundamental problem in drug discovery but challenging due to (i) the requirement of simultaneous optimization of multiple properties and (ii) the large chemical space to explore. Recently, deep learning methods have been proposed to solve this task by mimicking the chemist’s intuition in terms of matched molecular pairs (MMPs). Although MMPs is a widely used strategy by medicinal chemists, it offers limited capability in terms of exploring the space of structural modifications, therefore does not cover the complete space of solutions. Often more general transformations beyond the nature of MMPs are feasible and/or necessary, *e*.*g*. simultaneous modifications of the starting molecule at different places including the core scaffold. This study aims to provide a general methodology that offers more general structural modifications beyond MMPs. In particular, the same Transformer architecture is trained on different datasets. These datasets consist of a set of molecular pairs which reflect different types of transformations. Beyond MMP transformation, datasets reflecting general structural changes are constructed from ChEMBL based on two approaches: Tanimoto similarity (allows for multiple modifications) and scaffold matching (allows for multiple modifications but keep the scaffold constant) respectively. We investigate how the model behavior can be altered by tailoring the dataset while using the same model architecture. Our results show that the models trained on differently prepared datasets transform a given starting molecule in a way that it reflects the nature of the dataset used for training the model. These models could complement each other and unlock the capability for the chemists to pursue different options for improving a starting molecule.

**Supplementary Information:**

The online version contains supplementary material available at 10.1186/s13321-022-00599-3.

## Introduction

Molecular optimization aims to improve the property profile of a starting molecule. It plays an important role in the drug discovery and development process. However, this problem is challenging due to (i) the requirement of simultaneous optimization of multiple, often conflicting properties, *e*.*g*. physicochemical properties, ADMET (absorption, distribution, metabolism, elimination and toxicity) properties, safety and potency against its target and (ii) the large chemical space [1] to explore. Traditionally, chemists use their knowledge, experience and intuition [2] to apply chemical transformations to the starting molecule, to design improved molecules that have a balance of multiple properties. However, it heavily relies on chemist’s knowledge and is often impacted by individual‘s biases. This can limit the design process and the opportunities to find improved molecules within a reasonable time scale.

Recently, various deep learning methods have been used and proposed for *de novo* molecular design, *e*.*g*. recurrent neural networks (RNNs) [3–5], variational autoencoders (VAEs) [6–11] and generative adversarial networks (GANs) [12–15]. To improve the generated molecules towards desirable properties, reinforcement learning [12, 13, 15, 16], adversarial training [17–19], transfer learning [3] and different optimization techniques [6, 20] have been used. Conditional generative models [8, 11, 21, 22] have also been proposed where the desirable properties are incorporated as condition to directly control the generating process. However, most of them focus on generating molecules from scratch. There are only a few studies on generating molecules with desirable properties from a given starting molecule, which aim to solve the molecular optimization task directly. Most of them use a set of molecular pairs for training. Jin *et al.* [17, 23, 24] utilized molecular graph representations and viewed the molecular optimization problem as a graph-to-graph translation problem. He *et al.* [25, 26] instead utilized the string-based representation, the simplified molecular-input line-entry system (SMILES) [27] and employed the machine translation models [28, 29] from natural language processing (NLP). They trained machine translation models (Transformer and Seq2Seq) to mimic the chemist’s approach of using MMPs [30, 31] where two molecules differ by a single chemical transformation. It was shown that the Transformer performs better than the Seq2Seq and HierG2G architectures [24].

Application of MMPs is a widely used design strategy by medicinal chemists due to its interpretable and intuitive nature. However, MMPs are inherently limited in terms of structural modifications relevant for molecular optimization. From chemist’s perspective, there could be need for transformations that extend beyond the reach and capabilities of MMPs, such as simultaneous modifications of the molecule at multiple points or modifications of the core scaffold. Moreover, such modifications are often needed to reach the optimization goals. In this study, the same Transformer architecture is trained on different datasets. These datasets consist of a set of molecular pairs, and are prepared to reflect different types of transformations. To capture more general transformations beyond MMPs, two approaches are used to extract molecular pairs from ChEMBL: Tanimoto similarity (allows for multiple modifications) and scaffold matching [32] (allows for multiple modifications but keeps the scaffold constant) respectively. The goal of this study is not necessarily to benchmark against MMPs but instead to provide more general structural modifications than only MMPs. This could unlock the capability for the chemists to pursue different options for improving a starting molecule.

## Methods

**Fig. 1:**
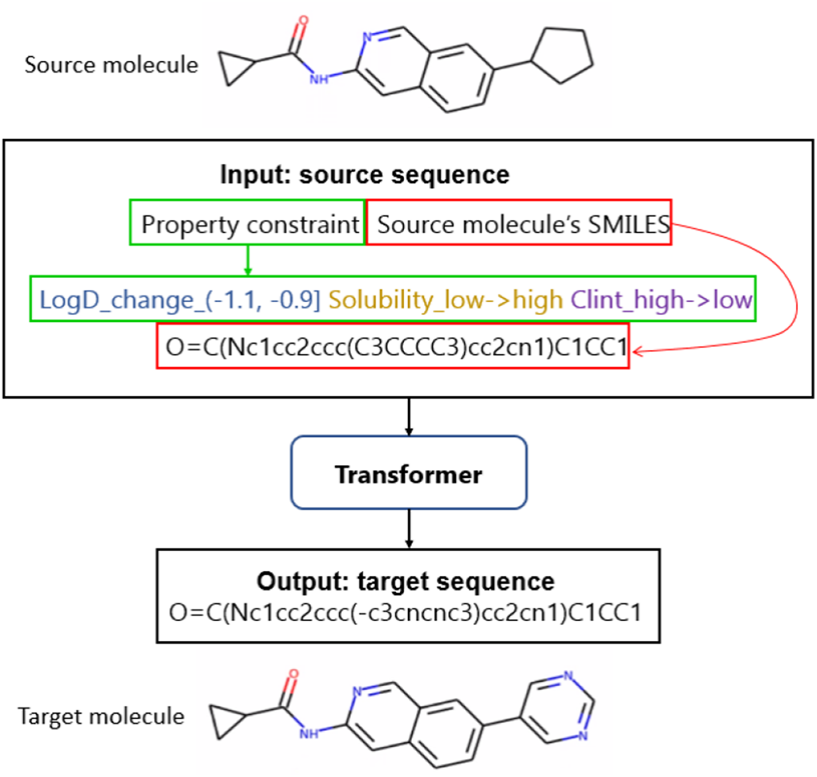
Input and output of the Transformer model (following [25]). The input is the concatenation of property change tokens and the SMILES of the starting molecule. During training, the output is the target molecule with the desirable properties while during inference the output is generated token by token and is expected to satisfy the property constraint in the input

Following [25], the SMILES representation of molecule and the Transformer model from NLP are used in our study. The Transformer model is trained on a set of molecular pairs together with the property changes between source and target molecules. Figure 1 shows an example of source and target sequences which are fed into the Transformer model. The input consists of property constraint and source molecule’s SMILES. The property constraint specifies how to change the source molecule.

Given a set of molecular pairs $\{(X, Y, Z)\}$ where *X* represents source molecule, *Y* represents target molecule, and *Z* represents the property change between source molecule *X* and target molecule *Y*, the Transformer model will learn a mapping $(X,Z)\in \mathcal {X}\times \mathcal {Z}\rightarrow Y\in \mathcal {Y}$ during training where $\mathcal {X}\times \mathcal {Z}$ represents the input space and $\mathcal {Y}$ represents the target space. During testing, given a new $(X,Z)\in \mathcal {X}\times \mathcal {Z}$, the model will be expected to generate a diverse set of target molecules with desirable properties [25].

**Table 1:** Property change encoding

Property	Measured unit	Threshold	Threshold in $log_{10}$ scale	Designed property change tokens
LogD	-	-	-	LogD_change_(− inf, − 6.9]
				...
				LogD_change_(− 0.3, − 0.1]
				LogD_change_(− 0.1, 0.1]
				LogD_change_(0.1, 0.3]
				...
				LogD_change_(6.9, inf]
Solubility	$\mu$M	low: $\le$50	low: $\le$1.7	Solubility_low$\rightarrow$high
		high: >50	high: >1.7	Solubility_high$\rightarrow$low
				Solubility_no_change
Clearance	$\mu$L/min/mg	low: $\le$20	low: $\le$1.3	Clearance_low$\rightarrow$high
		high: >20	high: >1.3	Clearance_high$\rightarrow$low
				Clearance_no_change

### Properties optimized

Three ADMET properties, *logD*, *solubility* and *clearance* which are important properties of a drug are selected to be optimized simultaneously. *LogD* is measured as a compound’s distribution coefficient between octanol and water at pH 7.4, based on the shake flask approach. *Solubility* is measured by the generation of a saturated solution of the compound, followed by assaying the solution using high-performance liquid chromatography (HPLC) with ultra violet (UV) quantification and mass spectrometry (MS) identification. The measured unit of *solubility* is $\mu$M. For *clearance*, human liver microsome intrinsic clearance (HLM CLint) is measured, and the unit is $\mu$L/min/mg. The measured in-house property data was used to build the property prediction models. These models were then applied to the processed molecules in ChEMBL to derive the data used for training the Transformer model. They are also used to estimate the properties of the generated molecules from the model. Details can be found in Section ADMET Property Prediction Model.

### Tokenizing SMILES and property changes

The Transformer model takes a sequence of tokens as input. Therefore the SMILES and property changes need to be tokenized to be recognized by the model. The SMILES is tokenized based on a single character with the exception of two-character tokens (*i*.*e*. , “Cl” and “Br”) and tokens between brackets (*e*.*g*. “[nH]” and “[O-]”). The tokenization was performed independently for each dataset.

Considering practical desirable criteria and experimental errors, *solubility* and *clearance* changes are encoded using three categories, while the change in *logD* is encoded into range intervals, with each interval length=0.2 except for the two open intervals on the sides (Table 1). The threshold for low/high *solubility* is 50 $\mu$M (1.7 in $log_{10}$ scale), and the threshold for low/high *clearance* is 20 $\mu$L/min/mg respectively (1.3 in $log_{10}$ scale). These property change tokens can be derived from the given input molecule’s properties and the target desirable properties. For example, if an input molecule’s *solubility* value is 10 $\mu$M and the target desirable solubility value is 80 $\mu$M, then the encoded property change token would be “Solubility_low$\rightarrow$ high”.

The vocabulary consists of all the tokens after performing the tokenization on all the SMILES and property changes of the molecular pairs in a dataset. Additionally, special tokens, *start* and *end* are added to signal the beginning and ending of a sequence.

### Transformer neural network

The same Transformer neural network in [25, 29] is used in this study. The Transformer consists of an encoder and a decoder. The network takes a sequence of tokens as input. Each token is converted into an embedding vector–a numerical representation of the token that can be processed by the network. The input tokens are fed into the network simultaneously. To capture the order information of the input tokens, positional encoding is performed on the embedding vectors. The resulting vectors are then passed through the encoder. The encoder is a stack of encoder layers, which process their input iteratively one layer after another. Each encoder layer converts its input (a sequence of vectors) into another sequence of vectors called encodings. These encodings are passed to the next encoder layer as input. The decoder is a stack of decoder layers of the same number as encoder. It does the opposite of the encoder: convert the encoder encodings into a sequence of tokens one token at a time. The attention mechanism is utilized in both encoder and decoder to encode or decode a current vector considering the importance of other vectors in the sequence. More details about the Transformer architecture can be found in [25, 29].

### Model training and sampling

The same Transformer architecture was trained with each dataset. Each model was trained on a single GPU (either NVIDIA GeForce RTX 2080 Ti or NVIDIA Tesla K80). The hyperparameters were set the same as [25]. The models were trained using a batch size of 128, Adam optimizer and the original learning rate schedule [29] with 4000 warmup steps. More details about the hyperparameters can be found in Additional file 1: Table S1.

After training, the model can be used to generate sequences given an input sequence. The sequence of tokens are generated one token at a time. At the first time step, the decoder takes the *start* token together with the encoder outputs as input, and samples an output token from the produced probability distribution over all the tokens in the vocabulary. The next time step will take all previous generated tokens and the encoder outputs as input. This process will continue until the *end* token is generated or a pre-defined maximum length of sequence is reached. To allow for the generation of multiple sequences, multinomial sampling is used.

### Data preparation

The datasets^1^ consist of a set of molecular pairs extracted from ChEMBL 28 [33]. In particular, the pairs were extracted from the molecules that are originated from the same publication since the molecules are more likely to be in the same project. Therefore, the molecular pairs are more likely to reflect the chemist’s intuition. The molecules, publications and molecular pairs are processed in the following fashion,

**Molecule pre-processing**

- Standardization using MolVS 2: Keep uncharged version of the largest fragment; Sanitize; RemoveHs; Disconnect metals; Apply normalization rules; Reionize acids; Keep sterochemistry
- 10 $\le$ Number of heavy atoms $\le$ 50
- Number of rings > 0
- AZFilter=“CORE” [34] to filter out low-quality compounds
- Substructure filters [35] for hit triaging with SeverityScore<10 3.
- Each molecule’s property values are within 3 standard deviations of all molecules’ property values (predicted)

**Publication pre-processing**

- Year $$\ge$$ 2000
- 10 $\le$ Number of molecules $\le$ 60

**Molecular pair pre-processing**

- Remove duplicated pairs (keep the earliest reported)
- Include reverse pairs

The resulting statistics on the data after performing the steps above can be found in Additional file 1: Figure S1.

#### Constructing molecular pairs

To capture different types of transformations, the following criteria are considered for extracting the pairs from different perspectives.

*MMP.* The matched molecular pairs are two molecules differ by a single transformation, which has been widely used as a strategy by medicinal chemists to support molecular optimization. Here, the MMPs are extracted using mmpdb, an open-source matched molecular pair tool [36]. The ratio between the number of heavy atoms (non-hydrogen atoms) in the R-group and the number of heavy atoms in the entire molecule is not greater than 0.33 [37].

**Fig. 2:**
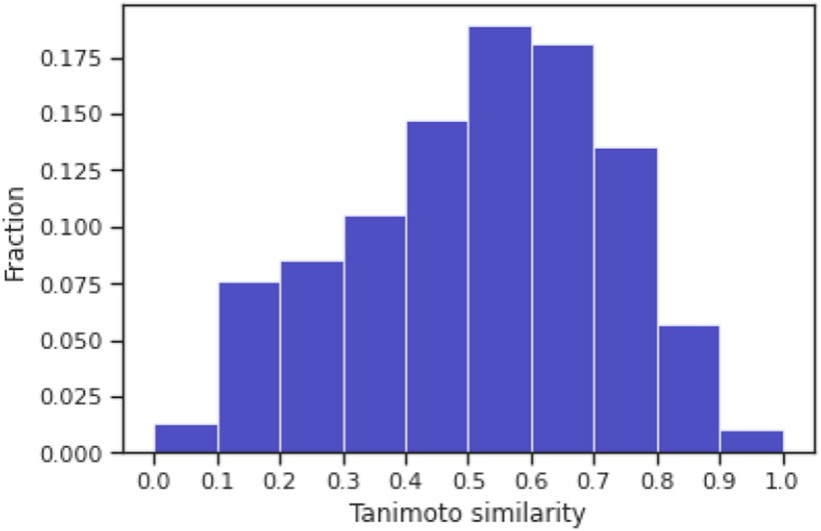
Tanimoto similarity distribution considering all the possible unique pairs with the same publication

To capture more general transformations (*e*.*g*. multiple modifications), apart from single transformations, the following criteria are used,

*Tanimoto similarity.* The Tanimoto similarity is computed based on Morgan Fingerprint with radius=2 (ECFP4) using RDKit. Figure 2 shows the distribution of Tanimoto similarity between all the possible unique pairs originating from the same publication. We extract the molecular pairs based on the following thresholds,

- Similarity ($\ge$0.5) for similar molecules
- Similarity ([0.5,0.7)) for medium similar molecules
- Similarity ($\ge$0.7) for highly similar molecules

*Scaffold matching.* For the molecules originating from the same publication, if two molecules share the same scaffold then they are extracted as pairs. In particular, the Murcko scaffold from RDKit which removes the side chains and the Murcko scaffold generic which converts all atom types to C and all bonds to single are used. The top 20 frequently occurring scaffold and generic scaffold can be found in Additional file 1: Figures S2 and S3.

Table 2 shows the resulting datasets (all datasets include reverse pairs). The training, validation and test sets are split based on the year of the publications from which the pairs are extracted. The Transformer neural network is trained on each dataset, and is expected to transform the input molecule in a way that it reflects the nature of the dataset used for training the model.

**Table 2:** Dataset

Datasets	Training (2000-2017)	Validation (2018)	Test (2019-2020)
MMPs	2,287,588	143,978	166,582
Similarity ($\ge$0.5)	6,543,684	418,180	475,070
Similarity ([0.5,0.7))	4,543,472	286,682	327,606
Similarity ($\ge$0.7)	2,000,212	131,498	147,464
Scaffold	2,850,180	171,914	199,786
Scaffold generic	4,127,058	255,580	289,034

#### ADMET property prediction model

The input of our Transformer model takes the property changes of molecular pairs into account. The property predictive models were built by using a message passing neural network [38]. Since the public data in ChEMBL on the properties of interest was scarce, we resorted to using in-house data instead. The solubility and clearance data are transformed to $log_{10}$ scale. The resulting models were used as a source of ground truth for deriving the training data. They were also used for evaluating the properties of the output from the Transformer model. Experimental verification would have been an expensive alternative and for the illustrative purposes of our work, we found that a simulated alternative of a wet lab experiment would be sufficient. Table 3 shows the train and test size, root-mean-square error (RMSE), normalized RMSE (NRMSE) and $R^2$ for each property prediction model.

**Table 3:** Property prediction model performance on in-house data

	LogD	Solubility	Clearance
Train size	186,575	197,988	155,652
Train RMSE	0.295	0.489	0.271
Train NRMSE	0.025	0.056	0.053
Train $R^2$	0.942	0.775	0.760
Test size	20,731	21,999	17,295
Test RMSE	0.395	0.600	0.352
Test NRMSE	0.038	0.076	0.091
Test $R^2$	0.897	0.659	0.555

### Experimental settings

For each starting molecule in the test set, 10 unique valid molecules, which are different from the starting molecule, were generated using multinomial sampling.

#### Evaluation metrics

The models are evaluated in two main aspects,

- **Successful property constraints** gives the percentage of generated molecules that fulfill the three desirable properties specified by model input simultaneously. The ADMET property prediction model in Table 3 is used to compute the properties of generated molecules. Following [25], the model error (Test RMSE in Table 3) is considered to determine if a generated molecule satisfies its desirable properties. For *logD*, the generated molecules with $|{{logD}}_{generated}-{{logD}}_{target}|\le 0.4$ will be considered as satisfying desirable *logD* constraint. For *solubility*, the threshold for low and high will be a range considering the model error, *i*.*e*. 1.7±0.6. The generated molecules with ${{solubility}}\le 2.3$ will be considered as low, and those with ${{solubility}}\ge 1.1$ will be considered as high. Similarly, for *clearance*, the threshold is 1.3±0.35.
- **Successful structure constraints** gives the percentage of generated molecules that when comparing with their corresponding starting molecules, have the same structure constraints as the pairs in the training set. This differs according to datasets, *e*.*g*. for the MMPs dataset, this metric gives the percentage of generated molecules that are matched molecular pairs with their starting molecules while for the Similarity ($\ge$0.5) dataset, the structure constraint is that the Tanimoto similarity between the generated molecules and their corresponding starting molecules is between 0.5 and 1.0. This metric evaluates if the model has learned to use the type of transformation reflected in the training set to modify starting molecules.

#### Baselines

We compare our model Transformer with the following baselines,

- **Transformer-U** is the unconditional Transformer architecture trained on molecular pairs but without any input property constraints.
- **Random** randomly selects 10 molecules (for a direct comparison with our Transformer model where 10 molecules are generated) from the unique set of molecules in the test set that have the same structure constraint as the training set. For example, for the Scaffold dataset, it randomly select 10 molecules that share the same scaffold with the given starting molecule. Since it is computationally expensive to evaluate all the samples (each sample consist of a starting molecule desirable property changes) in the test set, we randomly select 1% of the test set, repeat 5 times with different sampling seeds and report the average results. Note the Random baseline will always give 100% successful structure constraints due to its nature of fulfilling the structure constraints.

## Results and discussion

### Data statistics

**Fig. 3:**
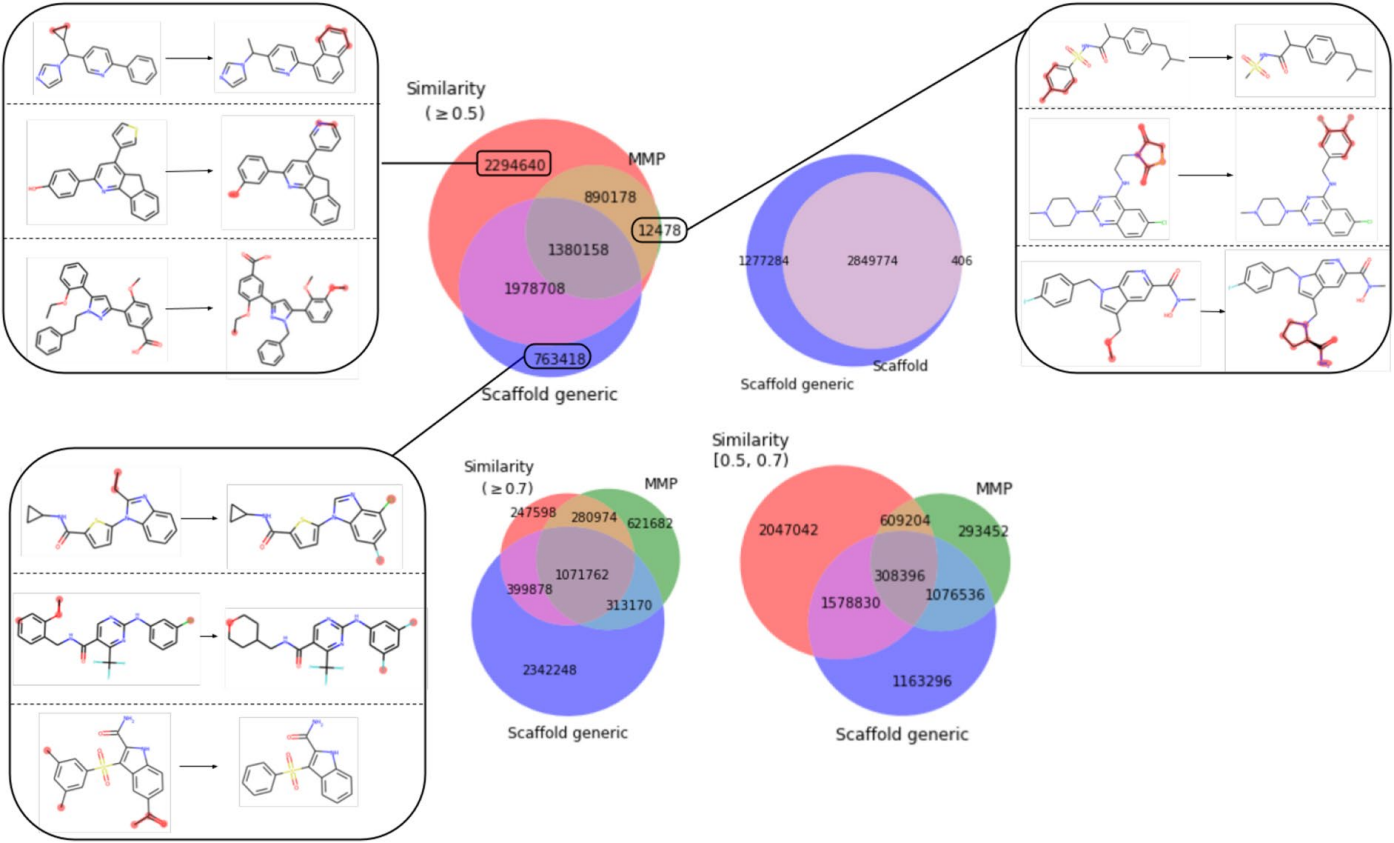
Overlap of training molecular pairs among different datasets. Exemplar molecular pairs are shown for data only in dataset Similarity ($\ge$0.5), scaffold generic and MMP respectively

Figure 3 shows the overlap of training molecular pairs among different datasets. Almost all the MMPs are in the dataset of pairs with Similarity ($\ge$0.5). The overlap between the MMP dataset and the Similarity ($\ge$0.7) dataset is bigger than the one between the MMP dataset and the Similarity ([0.5,0.7)) dataset. Exemplar molecular pairs only in dataset Similarity ($\ge$0.5) show that the scaffold is changed compared to pairs sharing generic scaffold and are non-MMPs because of multiple modifications and/or big change in R-group. The molecular pairs only in scaffold generic have Tanimoto similarity below 0.5. A tiny proportion of MMPs have Tanimoto similarity below 0.5 and change the scaffold.

**Table 4:** Performance comparison of Transformer and baselines in terms of successful property constraints, successful structure constraints and both metrics simultaneously

Dataset	Model	Successful property constraints (%)	Successful structure constraints (%)	Successful property and structure constraints (%)
MMP	Transformer	**61.90**	91.55	**58.09**
	Transformer-U	33.67	93.25	31.85
	Random	13.44±0.43	100	13.44±0.43
Similarity ($\ge$0.5)	Transformer	**51.83**	82.30	**44.53**
	Transformer-U	29.04	83.63	25.32
	Random	15.17±0.27	100	15.17±0.27
Similarity ([0.5,0.7))	Transformer	**46.75**	68.09	**32.96**
	Transformer-U	26.23	69.13	18.72
	Random	14.57±0.37	100	14.57±0.37
Similarity ($\ge$0.7)	Transformer	**65.09**	82.68	**56.07**
	Transformer-U	39.57	84.83	34.70
	Random	11.48±0.29	100	11.48±0.29
Scaffold	Transformer	**61.53**	95.32	**59.69**
	Transformer-U	37.16	95.69	36.26
	Random	17.22±0.74	100	17.22±0.74
Scaffold generic	Transformer	**55.05**	96.01	**53.66**
	Transformer-U	32.55	96.30	31.69
	Random	16.48±0.41	100	16.48±0.41

The results in bold indicate the best values; higher values are better

Each model is trained on the corresponding dataset for that row

**Fig. 4:**
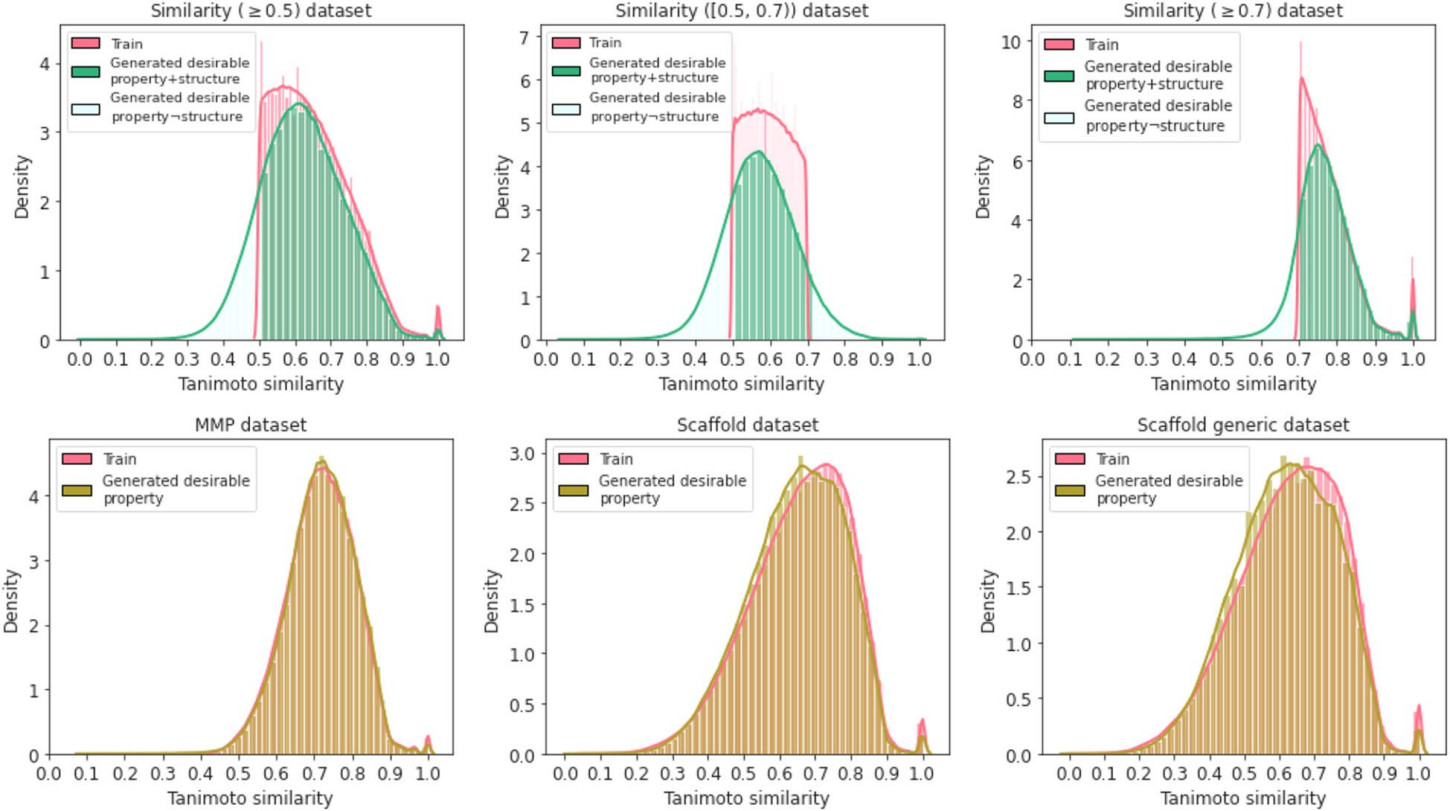
Tanimoto similarity distribution for Similarity (≥ 0.5) dataset, Similarity ([0.5,0.7)) dataset, Similarity (≥ 0.7) dataset, MMP dataset, Scaffold dataset and Scaffold generic dataset. Legend Train for the molecular pairs from the training set; Generated desirable property for the pairs between the generated molecules that fulfil successful property constraints and their starting molecules from the test set; Generated desirable property+structure for the pairs between the generated molecules that fulfil both successful property and structure constraints and their starting molecules from the test set; Generated desirable property$\lnot$structure for the pairs between the generated molecules that fulfil successful property but not structure constraints and their starting molecules from the test set

### Performance comparison with baselines

Table 4 compares our Transformer model with the baselines (Transformer-U and Random) in terms of successful property and structure constraints on different datasets. Transformer outperforms Transformer-U and Random in terms of successful property constraints, generating more molecules with desirable properties on all datasets. For the successful structure constraints, Transformer-U is comparable or better than Transformer. Transformer-U has learned to generate “similar” molecules to the given input starting molecules. However, it generates much less molecules with desirable properties compared to Transformer. It is mainly because Transformer-U was trained only on molecular pairs, and does not include the property change of the pairs in the input, while Transformer having the property changes as additional input, allows for more directed output generation. Both Transformer and Transformer-U outperform the Random baseline—finding more molecules that satisfy desirable properties and structure constraint simultaneously.

**Table 5:** Performance comparison of the Transformer models trained on different types of molecular pairs on the restricted intersection test set (numbers in bracket represent the absolute increase or decrease compared to the corresponding Transformer model performance on the original test set in Table 4)

Test set	Type of molecular pairs where Transformer is trained	Successful property constraints (%)	Successful structure constraints (%)	Successful property and structure constraints (%)
	MMP	**65.71** ($\uparrow$ 3.81)	91.68 ($\uparrow$ 0.13)	**61.82** ($\uparrow$ 3.73)
	Similarity ($\ge$0.5)	55.55 ($\uparrow$ 3.72)	84.47 ($\uparrow$ **2.17**)	48.97 ($\uparrow$ **4.44**)
Restricted	Similarity ([0.5,0.7))	**50.17** ($\uparrow$ 3.42)	**68.66** ($\uparrow$ 0.57)	**35.28** ($\uparrow$ 2.32)
intersection	Similarity ($\ge$0.7)	65.39 ($\uparrow$ **0.30**)	81.49 ($\downarrow$ **1.19**)	55.55 ($\downarrow$ 0.52)
	Scaffold	62.91 ($\uparrow$ 1.38)	94.42 ($\downarrow$ 0.90)	60.70 ($\downarrow$ **1.01**)
	Scaffold generic	59.07 ($\uparrow$ **4.02**)	**96.14** ($\uparrow$ 0.13)	57.68 ($\uparrow$ 4.02)

The extremes (best/worst performance or largest/smallest change) are highlighted in bold

Figure 4 compares the Tanimoto similarity distribution of the molecular pairs from the training set with the one between the generated molecules and their starting molecules from the test set for the Transformer model. It can be seen that the distribution of the generated pairs align well with the pairs from the training set for most datasets. This indicates that the model has learned to transform a given starting molecule in a way that it reflects the nature of the training data. For the datasets based on Tanimoto similarity, the alignment is worse, but the model systematically generates molecules that fulfil the successful property constraints. This can be seen from the areas (lightcyan) that are outside the constrains of the training set (red). This also indicates the model can extrapolate the learning beyond the structure constraints defined by the training data. Additionally, the overlap between the Tanimoto similarity distribution of molecular pairs from the training set (red) and the one from the test set (yellow) for the scaffold-based datasets is slightly worse than the overlap for the MMP dataset in Fig. 4. However, from Table 4, the models trained on scaffold-based datasets perform better than the one trained on the MMP dataset in terms of fulfilling successful structure constraints. This might be because the structural changes with MMPs are in general smaller than the ones with scaffold-based pairs, which tends to keep the Tanimoto similarity higher. On the other hand, it is relatively easy for the model trained on molecular pairs sharing the same scaffold to maintain the same scaffold while introducing multiple modifications. For the model trained on MMPs, the modification has to be a single and a small transformation in order to fulfill the successful structure constraint.

### Performance comparison of models trained on different types of molecular pairs

With the following experiments, we evaluate how the models trained on different types of molecular pairs perform on the same test sets. Table 5 shows the results on the restricted intersection test set which is the intersection of MMP, Similarity ($\ge$0.5) and Scaffold generic test sets. Details about the test sets, and the results for other test sets can be found in Additional file 1 (p.6-7).

The model trained on MMP dataset performs best in terms of successful property constraints, followed closely by the one trained on Similarity ($\ge$0.7) dataset, while the model trained on Similarity ([0.5, 0.7)) dataset performs worst. This might be because the molecular pairs in the restricted intersection test set have smaller structural changes and desired property changes, and it is easier to achieve small desirable property changes by making small structural changes. It might also be because of the varying performance of the models trained on different types of molecular pairs in the beginning (Table 4). Therefore we also report the difference (numbers in bracket) compared to their performance on their original test sets (Table 4). We can see that most models perform better compared to the performance on their own original test set, indicating this restricted intersection test set is an relative easy task. The performance change of the models trained on Similarity ($\ge$0.7) and Scaffold are very small, indicating there is not much difference between this restricted dataset and their own original test set in terms of difficulty.

**Fig. 5:**
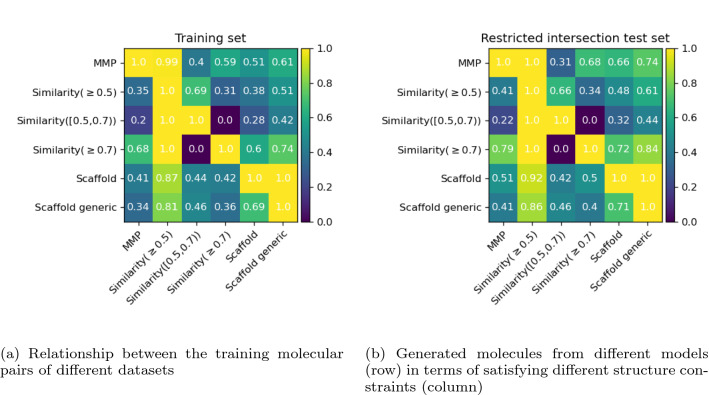
Comparison of heatmaps for training set and test set. The more similar, the better. **a** Relationship between the training molecular pairs of different datasets, *e*.*g*. the number 0.2 with Similarity ([0.5, 0.7)) as row and MMP as column from the training set represents 20% of the pairs with Similarity ([0.5, 0.7)) are also MMPs. **b** Each row represents the model trained on the corresponding dataset, and each column represents the corresponding structure constraints. The number 0.22 with Similarity ([0.5, 0.7)) as row and MMP as column from the Restricted intersection test set represents that when looking at the generated molecules using the Transformer model trained on Similarity ([0.5, 0.7)) dataset, among all the ones fulfilling the the property constraints and structure constraints (*i*.*e*. Similarity ([0.5, 0.7))), 22% of them are MMPs. The diagonal for the Restricted intersection is always 1 because we only look at the generated molecules that already fulfil the property constraints and structure constraints

Figure 5a shows how the training molecular pairs from different datasets correlate with each other. For example, 40% of MMPs (row) are also pairs with Similarity ([0.5, 0.7)) (column) but only 20% of pairs with Similarity ([0.5, 0.7)) (row) are MMPs (column). Figure 5b shows that for the restricted intersection test set, how the generated molecules from models trained on different datasets satisfy different structure constraints. For example, among the generated molecules (that satisfy the property constraints and structure constraints, *i*.*e*. Similarity ([0.5, 0.7))) from the model trained on Similarity ([0.5, 0.7)) (row), 22% of them are MMPs when comparing with their corresponding starting molecules. Compared to the heatmap for the training set, the one for Restricted intersection test set basically follow the same pattern (similar patterns are found on other test sets), indicating the models have learned to modify the starting molecules in the way that it reflects the nature of the training set. Overall, it is shown that there is no single model generating molecules that cover the ones from all other models. It could be beneficial to use an ensemble of these models which complement each other to provide different options to transform a starting molecule towards desirable properties.

### Performance on test sets with large property changes desired

With the following experiments, we evaluate how the models trained on different types of molecular pairs perform on the test sets where large property changes (*logD* change is above 1; *solubility* and *clearance* change is either low$\rightarrow$high or high$\rightarrow$low) are desired. The molecular pairs in the original test sets where large property changes are extracted and merged excluding duplicates. Table 6 shows that 4.6% (highest) of the Similarity ([0.5, 0.7)) dataset has large property changes desired while Similarity ($\ge$0.7) dataset has the lowest, 2.3%. It is reasonable because it is less likely to have large property changes while keeping higher structural similarity.

**Table 6:** Test sets where big property changes (*logD* change is above 1; *solubility* and *clearance* change is either low$\rightarrow$high or high$\rightarrow$low) are desired

Test set	Size	Percentage (%)
MMP	6,180	3.7
Similarity ($\ge$0.5)	18,546	3.9
Similarity ([0.5, 0.7))	15,130	4.6
Similarity ($\ge$0.7)	3,416	2.3
Scaffold	6,252	3.1
Scaffold generic	10,514	3.6
Merged	21,652	-

Size indicates the number of data points where big property change are desired; Percentage indicates the fraction of the original test set in Table 2 with data points that have big property changes, *e*.*g*. 6180/166582$\approx$3.7%

**Table 7:** Performance comparison of Transformer models trained on different types of molecular pairs on the Merged dataset where big property changes are desired (numbers in bracket represent the absolute increase/decrease compared to the corresponding Transformer model performance on the original test set in Table 4)

Test set	Type of molecular pairs where Transformer is trained	Successful property constraints (%)	Successful structure constraints (%)	Successful property and structure constraints (%)
	MMP	**40.82** ($\downarrow$ 21.08)	83.89 ($\downarrow$ 7.66)	**36.12** ($\downarrow$ 21.97)
	Similarity ($\ge$0.5)	39.81 ($\downarrow$ 12.02)	75.00 ($\downarrow$ 7.30)	30.70 ($\downarrow$ 13.83)
Merged	Similarity ([0.5,0.7))	38.33 ($\downarrow$ **8.42**)	**66.64** ($\downarrow$ **1.45**)	25.94 ($\downarrow$ **7.02**)
	Similarity ($\ge$0.7)	**36.14** ($\downarrow$ **28.95**)	68.57 ($\downarrow$ **14.11**)	**25.58** ($\downarrow$ **30.49**)
	Scaffold	36.50 ($\downarrow$ 25.03)	89.17 ($\downarrow$ 6.15)	33.60 ($\downarrow$ 23.09)
	Scaffold generic	37.78 ($\downarrow$ 17.27)	**91.30** ($\downarrow$ 4.71)	35.26 ($\downarrow$ 18.40)

The extremes (best/worst performance or largest/smallest change) are highlighted in bold

**Fig. 6:**
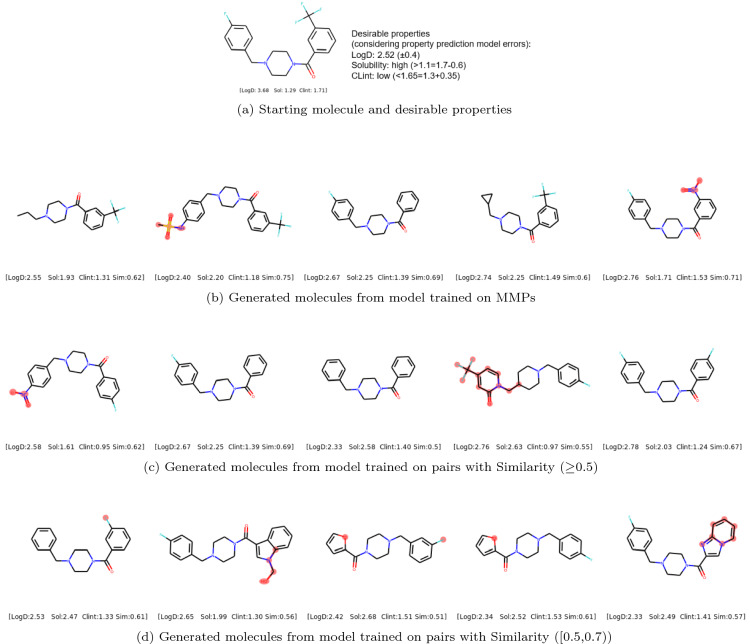
Example of diverse molecules with desirable properties generated by models trained on (**b**) MMPs (**c**) pairs with Similarity ($\ge$0.5) (**d**) pairs with Similarity ([0.5, 0.7)). The changes in the generated molecules compared with starting molecule are highlighted in red. Sim represents Tanimoto similarity

**Fig. 7:**
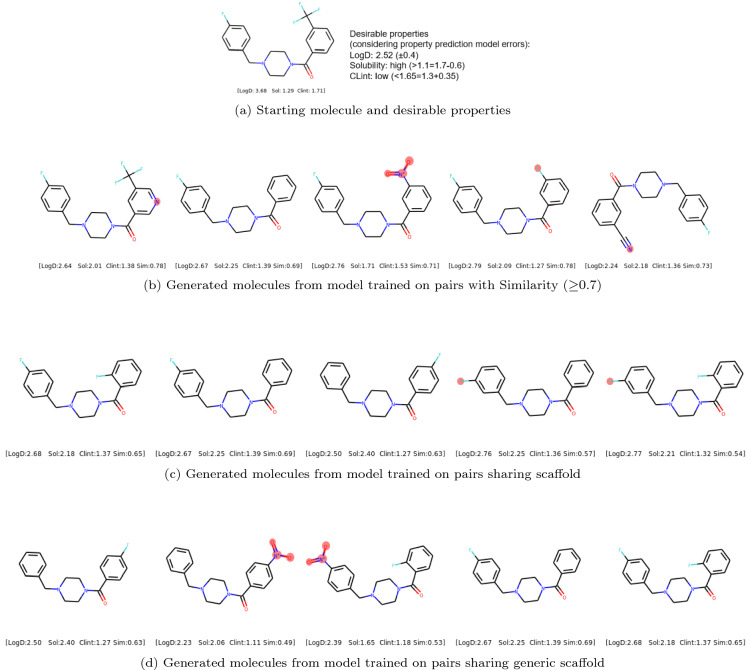
Example of diverse molecules with desirable properties generated by models trained on **b** pairs with Similarity ($\ge$0.7) **c** pairs sharing scaffold and **d** pairs sharing generic scaffold. The changes in the generated molecules compared with starting molecule are highlighted in red. Sim represents Tanimoto similarity

Table 7 shows the results on the merged dataset (the results on other datasets in Table 6 can be found in Additional file 1: Table S5). All models perform worse compared to their performance on their original test set (Table 4). The reason is that only a small proportion of molecular pairs having large property changes in the training set (Additional file 1: Figure S4), therefore the models generalize less well on such pairs. Intuitively, it would be expected that the model trained on Similarity ([0.5, 0.7)) dataset would perform best since it has higher percentage of pairs with large property changes for training and have more freedom to modify the starting molecule. However, it is observed that the model trained on MMPs performs best. This might be because it is easier to train the Transformer model for MMPs compared to pairs with similarity ([0.5, 0.7)) (already seen in Table 4) due to the smaller extrapolated space. Having that said, the performance of the models trained on different types of molecular pairs differ less on this Merged test set where big property changes are desired compared to previous test sets ( (Table 4 and Table 5). When looking at the numbers in bracket, we observed that the performance of model trained on Similarity ([0.5, 0.7)) drop the least, while the one for Similarity ($\ge$0.7) drop the most, followed by Scaffold and MMP.

### Example of diverse molecules generated using models trained on different types of molecular pairs

Figures 6 and 7 show an example of the generated molecules that fulfill the desirable properties but modify the starting molecule in different ways depending on the training data used for training the model. In particular, the generated molecules in Fig. 6b make a single transformation to the starting molecule while the ones in Fig. 7c and 7d allow for multiple modifications but keep the scaffold or generic scaffold constant. The generated molecules in Fig. 6c, 6d and 7b allow for multiple modifications and changes in scaffold, but the Tanimoto similarity lies approximately [0.5, 1.0], [0.7, 1.0] and [0.5, 0.7) respectively. Overall, this shows the flexibility of modifying starting molecules to achieve desirable properties in different ways by using the models trained on different types of molecular pairs.

### Discussion

#### Varying performance of models trained on different types of molecular pairs

The Transformer models trained on different datasets show varying performance as shown in Table 4. For the MMP, scaffold and scaffold generic datasets, it is easier to generate molecules in terms of successful structure constrains (MMPs, sharing same scaffold) compared to the datasets based on Tanimoto similarity split. This might be because the pairs in the Tanimoto similarity based datasets have more variations, and the models have more freedom to extrapolate which makes it difficult to keep the same structure constraints. It might also be due to the hard Tanimoto similarity cutoff used for constructing the training set (Fig. 4), which is difficult for the generated molecules from the Transformer model to follow on.

In terms of successful property constrains, Similarity ($\ge$0.7) dataset has the best performance, followed by MMP and scaffold, which are much better than Similarity ([0.5,0.7)), Similarity ($\ge$0.5) and scaffold generic. The reason might be that the extrapolated space is larger which makes it harder to find molecules with desirable properties. It might also be because the molecular pairs are more similar and the property changes are smaller for Similarity ($\ge$0.7), MMP and scaffold dataset (Additional file 1: Figure S4).

#### Varying performance in terms of successful structure constraints and successful property constraints

It is observed from Table 4 that the Transformer model’s performance in terms of successful structure constraints is better than successful property constraints. This might be because it is a relative easy task to keep the same structure constraint as in the training set. While for successful property constraints, it is more restricted due to the requirement of satisfying three properties simultaneously and the *logD* change is encoded at a higher level of granularity (considering the practical use) compared to *solubility* and *clearance* change which only have three possible changes (Table 1). This makes the input space more complicated and bigger, which requires more data to build a good model and makes it harder to generalize well.

#### Molecular optimization beyond MMPs

The goal of this study is not necessarily to benchmark against MMPs, but instead to provide a general methodology that enables general structural changes beyond what MMPs are designed for. The application of MMPs is a useful concept, but it poses a limitation of exploring a broader chemical space. Often structural modifications beyond the reach of MMPs are feasible and/or needed to reach optimization goals. The presented method and results deliver the opportunity of exploring a broader space of structural modifications for molecular optimization. There is an observed tendency that it is more challenging for the model to learn from the datasets with larger structural changes, *i*.*e*. Similarity ($\ge$0.5), Similarity ([0.5, 0.7)) and Scaffold generic. The reason might be because the navigated chemical space is larger and it is hard to relate the large structural changes to accurate property changes. Nevertheless, these models provide alternatives to MMPs, which is useful when MMPs are not adequate or feasible during optimization. This study shows how tailoring the training datasets can lead to the changes in the behaviour of the resulting trained model. This concept can be extrapolated to any user-specified structure modification.

## Conclusions

We propose a general methodology to provide more general structural transformations beyond MMPs for molecular optimization. This can be achieved by tailoring the dataset accordingly while using the same model architecture. Different types of dataset (molecular pairs) were extracted from ChEMBL based on MMPs, Tanimoto similarity and scaffold matching which result in six datasets: MMPs, Similarity ($\ge$0.5), Similarity ([0.5, 0.7)), Similarity ($\ge$0.7)), Scaffold and Scaffold generic. These datasets reflect different types of transformations, and the Transformer neural network was trained on each dataset. Our results showed that it is relatively easy to keep the structure constraints for MMP and Scaffold-based datasets compared to Tanimoto similarity-based datasets. Furthermore, the models trained on different types of molecular pairs transform a given starting molecule in a way that it reflects the nature of the dataset used for training the model, *e*.*g*. the model trained on MMPs modify the starting molecules by a single transformation, the models trained on similarity based molecular pairs allow for multiple modifications but keep the Tanimoto similarity in certain ranges, and the model trained on Scaffold-based molecular pairs allow for multiple modifications but keep the scaffold or generic scaffold constant. These models could complement each other and unlock the capability for the chemists to pursue different options for improving a starting molecule, therefore accelerate the drug discovery process.

## Supplementary Information

**Additional file 1: Table S1**: Hyperparameters for the Transformer model. **Table S2**. Training sets where big property changes (logD change is above 1; solubility and clearance change is either low→high or high→low) aredesired. Percentage indicates the fraction of training sets with data points that have big property changes. **Table S3**. Test sets extracted for model comparison. **Table S4**. Performance comparison of the Transformer models trained on different types of molecular pairs on different test sets (numbers in bracketrepresent the absolute increase or decrease compared to the corresponding Transformer model performance on the original test set in Table 4). Theextremes (best/worst performance or largest/smallest change) are highlighted in bold. **Table S5**. Performance comparison of Transformer models trained on different types of molecular pairs on different test sets where big propertychanges are desired (numbers in bracket represent the absolute increase/decrease compared to the corresponding Transformer model performanceon the original test set in Table 4). The extremes (best/worst performance or largest/smallest change) are highlighted in bold. **Figure S1**. Data statistics after performing the pre-processing steps (described in Data Preparation section) on the molecules and the publicationsavailable in ChEMBL 28. Publications Per Year: the number of publications published per year; Molecules Per Publication: the number of moleculesthat are released per publication. **Figure S2**. Top 20 frequently occurring scaffolds in the Scaffold training set. **Figure S3**. Top 20 frequently occurring generic scaffolds in the Scaffold generic training set. **Figure S4**. Property change distribution for different training datasets. Each tick in the horizontal axis represents the combination of logD, solubility and clint changes. For example, the first tick big change; high→ low; high→ low represents the logD change is big change, solubility change ishigh→ low, and clint change is high→ low. For logD change, no change includes (-0.1, 0.1]; small change includes changes below 0.5; medium change includes between 0.5 and 1; big change includes changes above 1. **Figure S5**. Overlap of molecular pairs among different test sets, MMP,Similarity (≥0.5), Scaffold generic datasets, used for extracting test sets for model comparison.

## Data Availability

All source code and datasets used to produce the reported results can be found at https://github.com/MolecularAI/deep-molecular-optimization/tree/general_transformation and https://doi.org/10.5281/zenodo.6319821.

## Abbreviations

MMPs: Matched molecular pairs
ADMET: Absorption, distribution, metabolism, elimination and toxicity
RNNs: Recurrent neural networks
VAEs: Variational autoencoders
GANs: Generative adversarial networks
SMILES: Simplified Molecular-Input Line-Entry System
NLP: Natural language processing
Seq2Seq: Sequence to sequence
HierG2G: Hierachical graph encoder-decoder
HPLC: High-performance liquid chromatography
UV: Ultra violet
MS: Mass spectrometry
HLM CLint: Human liver microsome intrinsic clearance
RMSE: Root-mean-square error
NRMSE: Normalized RMSE

## Footnotes

1 https://doi.org/10.5281/zenodo.6319821.

2 https://molvs.readthedocs.io/en/latest/.

3 https://github.com/rdkit/rdkit/tree/master/Contrib/NIBRSubstructureFilters.
