# Supplementary material for: Transformer-based molecular optimization beyond matched molecular pairs
Source: J Cheminform. 2022 Mar 28;14:18. doi: 10.1186/s13321-022-00599-3 (PMC8962145; doi:10.1186/s13321-022-00599-3)
Supplement: Supplementary file 1 — Additional file 1: Table S1: Hyperparameters for the Transformer model. Table S2. Training sets where big property changes (logD change is above 1; solubility and clearance change is either low→high or high→low) aredesired. Percentage indicates the fraction of training sets with data points that have big property changes. Table S3. Test sets extracted for model comparison. Table S4. Performance comparison of the Transformer models trained on different types of molecular pairs on different test sets (numbers in bracketrepresent the absolute increase or decrease compared to the corresponding Transformer model performance on the original test set in Table 4). Theextremes (best/worst performance or largest/smallest change) are highlighted in bold. Table S5. Performance comparison of Transformer models trained on different types of molecular pairs on different test sets where big propertychanges are desired (numbers in bracket represent the absolute increase/decrease compared to the corresponding Transformer model performanceon the original test set in Table 4). The extremes (best/worst performance or largest/smallest change) are highlighted in bold. Figure S1. Data statistics after performing the pre-processing steps (described in Data Preparation section) on the molecules and the publicationsavailable in ChEMBL 28. Publications Per Year: the number of publications published per year; Molecules Per Publication: the number of moleculesthat are released per publication. Figure S2. Top 20 frequently occurring scaffolds in the Scaffold training set. Figure S3. Top 20 frequently occurring generic scaffolds in the Scaffold generic training set. Figure S4. Property change distribution for different training datasets. Each tick in the horizontal axis represents the combination of logD, solubility and clint changes. For example, the first tick big change; high→ low; high→ low represents the logD change is big change, solubility change ishigh→ low, and clint change is high→ low. [file 13321_2022_599_MOESM1_ESM.pdf]

Transformer-Based Molecular Optimization Beyond Matched Molecular Pairs

Jiazhen He[1*], Eva Nittinger[2], Christian Tyrchan[2], Werngard Czechtizkyr[2],
Atanas Patronov[1], Esben Jannik Bjerrum[1], Ola Engkvist[1,3]

[1]*Molecular AI, Discovery Sciences, R&D, AstraZeneca, Gothenburg, Sweden*
[2]*Medicinal Chemistry, Research and Early Development, Respiratory and Immunology (R&I), BioPharmaceuticals R&D, AstraZeneca, Gothenburg, Sweden*
[3]*Department of Computer Science and Engineering, Chalmers University of Technology, Gothenburg, Sweden*
[*]*Correspondence: jiazhen.he@astrazeneca.com*

Supplementary material

Hyperparameter settings

Table S1: Hyperparameters for the Transformer model

Parameter	Value
Number of layers	6
Number of attention heads	8
Embedding dimension	256
Feed-forward network dimension	2048
Dropout	0.1
Label smoothing	0
Batch size	128
Optimizer	Adam
Adam beta 1	0.9
Adam beta 2	0.98
Adam eps	1e-9
Warmup steps	4000

Data Statistics

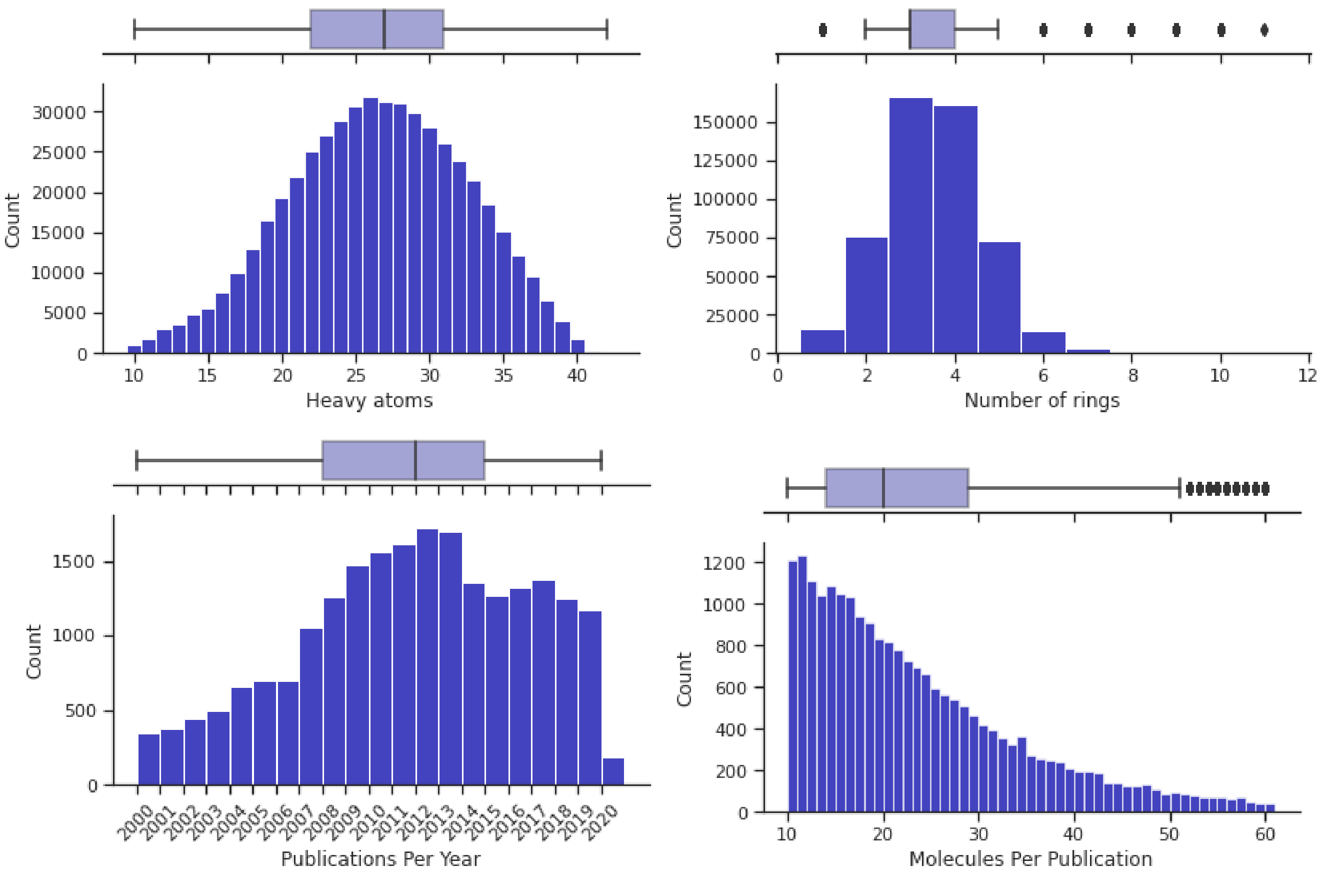

Figure S1: Data statistics after performing the pre-processing steps (described in Data Preparation section) on the molecules and the publications available in ChEMBL 28. Publications Per Year: the number of publications published per year; Molecules Per Publication: the number of molecules that are released per publication.

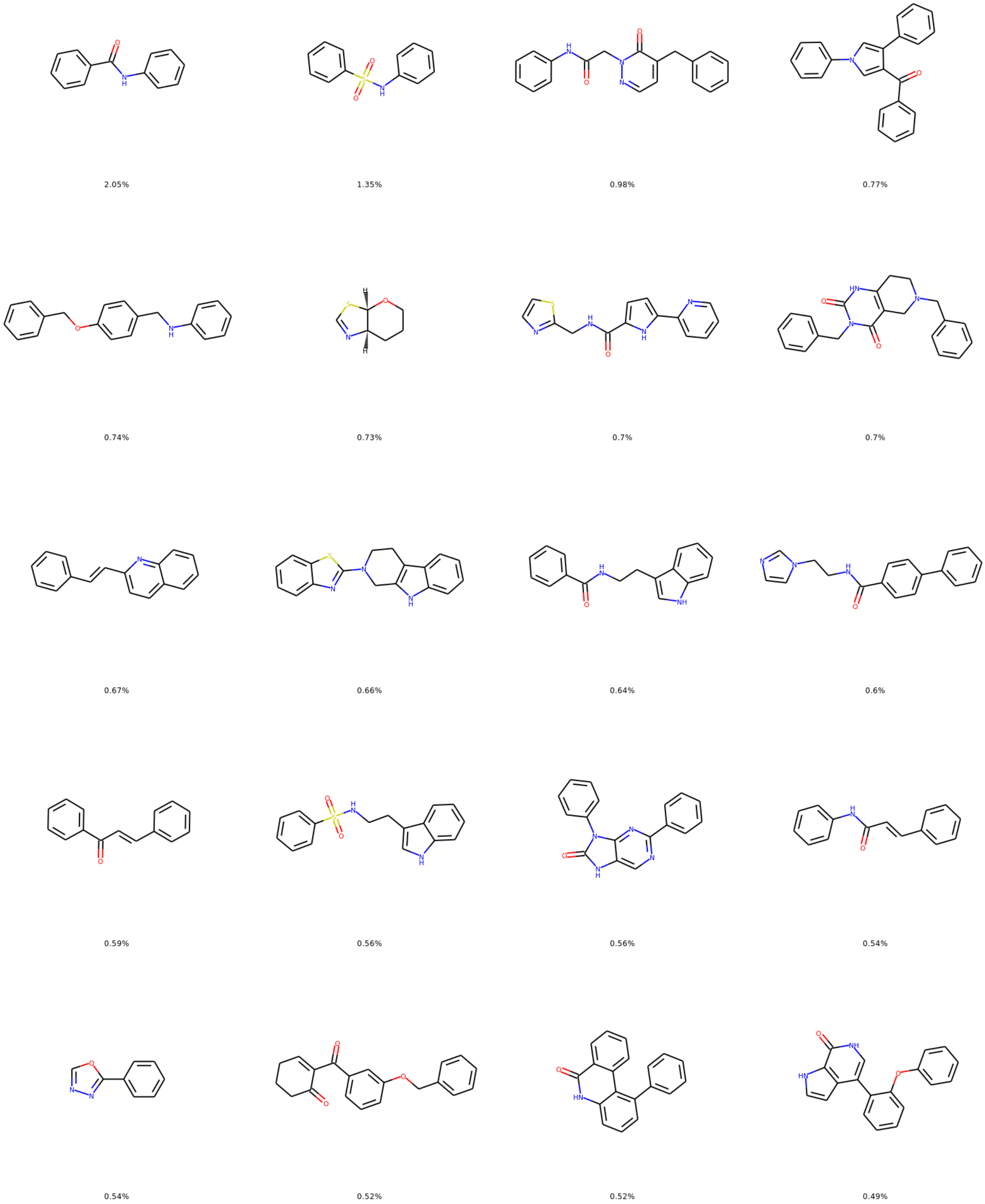

Figure S2: Top 20 frequently occurring scaffolds in the Scaffold training set.

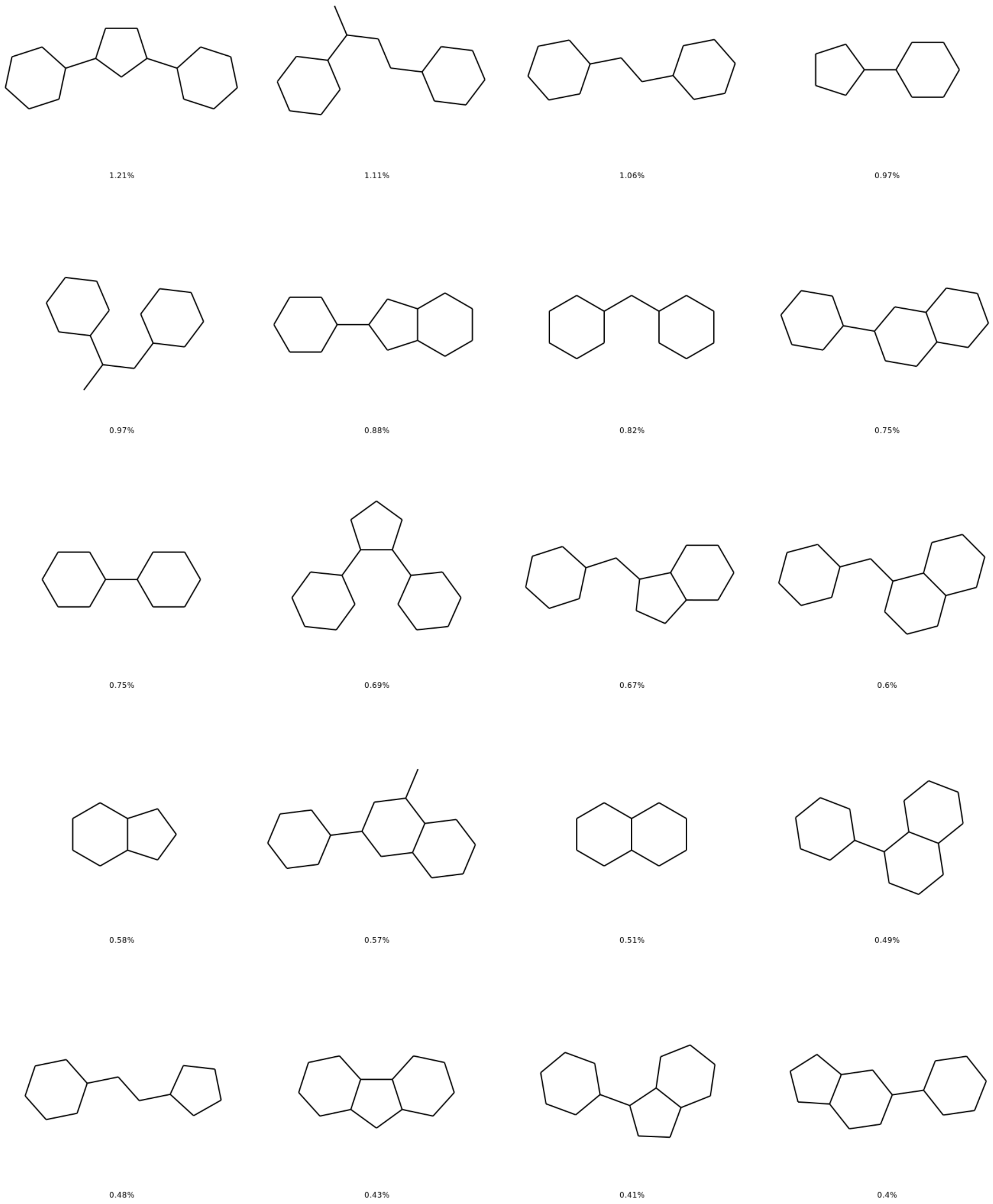

Figure S3: Top 20 frequently occurring generic scaffolds in the Scaffold generic training set.

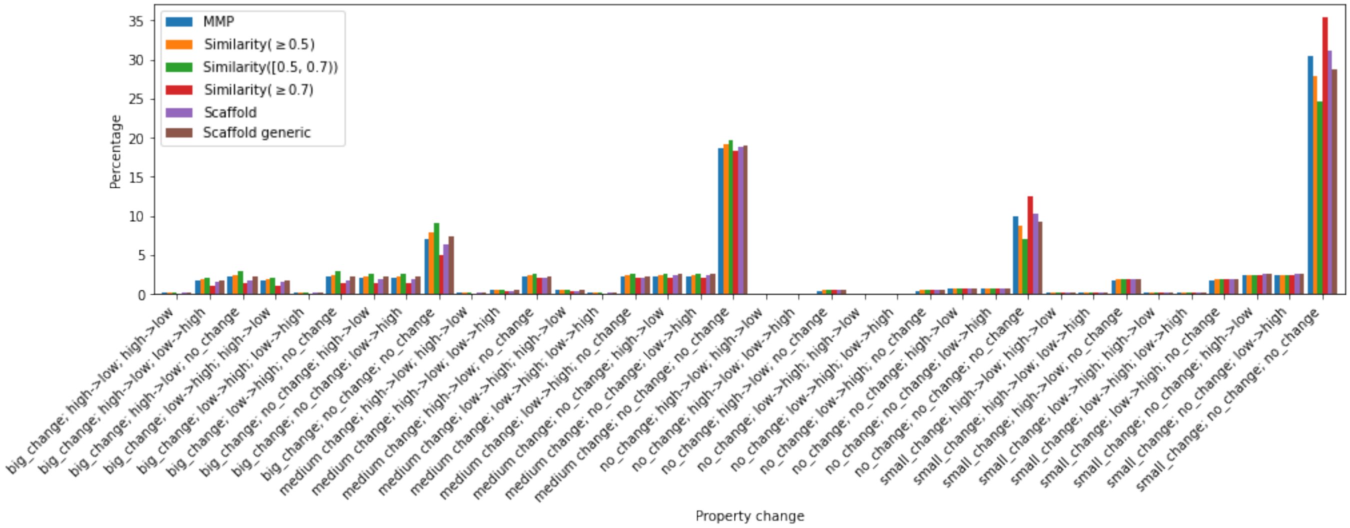

Figure S4: Property change distribution for different training datasets. Each tick in the horizontal axis represents the combination of logD, solubility and clint changes. For example, the first tick big_change; high→ low; high→ low represents the logD change is big_change, solubility change is high→ low, and clint change is high→ low. For logD change, no_change includes (-0.1, 0.1]; small_change includes changes below 0.5; medium change includes between 0.5 and 1; big_change includes changes above 1.

Table S2: Training sets where big property changes (*logD* change is above 1; *solubility* and *clearance* change is either low→high or high→low) are desired. Percentage indicates the fraction of training sets with data points that have big property changes.

Training set	Percentage (%)
MMP	3.7
Similarity (≥0.5)	3.9
Similarity ([0.5, 0.7))	4.7
Similarity (≥0.7)	2.2
Scaffold	3.4
Scaffold generic	3.7

Test sets extracted

Figure S5 shows the overlap of the original test sets (Table 2) among MMP, Similarity (≥0.5) and Scaffold generic test sets. Here, five test sets are extracted based on Figure S5 for model comparison, shown in Table S3.

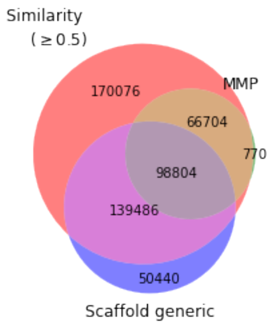

Figure S5: Overlap of molecular pairs among different test sets, MMP, Similarity (≥0.5), Scaffold generic datasets, used for extracting test sets for model comparison.

Table S3: Test sets extracted for model comparison. **Restricted intersection** represents the overlapping among MMP, Similarity (≥0.5) and Scaffold generic test sets; **Merged** represents the union of MMP, Similarity (≥0.5) and Scaffold generic test sets; **MMP only** represents the set of molecular pairs that appear only in MMP test set not the other two; Similarly for Similarity (≥0.5) only and Scaffold generic only.

Test set	Size
Restricted intersection	98,804
MMP only	770
Similarity (≥0.5) only	170,076
Scaffold generic only	50,440
Merged	526,584

Performance on the extracted test sets

Table S4 shows the model performance on the extracted test sets (Table S3). Unlike the performance on the restricted intersection dataset which mostly increases compared to the original test sets, the performance on the test sets–MMP only, Similarity (≥0.5) only and Scaffold generic only–drops significantly, indicating they are more difficult tasks than the original test sets. The performance of models trained on Similarity (≥0.7) and Scaffold drop the most (see bracket) in most cases. This might be because it is difficult to achieve desired properties by keeping high similarity or same scaffold. While for the model that trained on Similarity (≥0.5), it has the worst performance but drops the least in most cases. This might be because that the molecular pairs are less restrictive, but also make them more difficult to train. The performance of the model trained on MMP dataset dropped significantly on the MMP-only test set compared to the original full MMP test set (Table 4). We believe it is because the nature of the MMP-only test set: the data points are very different from the majority, *i.e.* the Similarity is below 0.5 and the generic scaffold is changed. And there is only 770 samples (Figure S5) on the MMP-only test set compared to 166,582 samples (Table 2) on the full test set. The model does not generalize well on such "corner" data points. On the other hand, the model performance improves on the restricted interaction test set (overlapping between MMP, Similarity≥0.5 and Scaffold generic) compared to the full MMP test set. These indicate the model can perform differently on different subsets of the full original test set. The performance on the merged test set lie between the one on the restricted intersection test set and the ones on other test sets. For the models trained on Similarity (≥0.5) and Similarity ([0.5, 0.7)), there is not much difference (see bracket) compared to the performance on their own original test set, while the models trained on Similarity (≥0.7) and Scaffold are more sensitive to the test sets.

Table S4: Performance comparison of the Transformer models trained on different types of molecular pairs on different test sets (numbers in bracket represent the absolute increase or decrease compared to the corresponding Transformer model performance on the original test set in Table 4). The extremes (best/worst performance or largest/smallest change) are highlighted in bold.

Test set	Type of molecular pairs where Transformer is trained	Successful property constraints (%)	Successful structure constraints (%)	Successful property and structure constraints (%)
	MMP	**65.71** (↑ 3.81)	91.68 (↑ 0.13)	**61.82** (↑ 3.73)
	Similarity (≥0.5)	55.55 (↑ 3.72)	84.47 (↑ **2.17**)	48.97 (↑ **4.44**)
Restricted	Similarity ([0.5,0.7))	**50.17** (↑ 3.42)	**68.66** (↑ 0.57)	**35.28** (↑ 2.32)
intersection	Similarity (≥0.7)	65.39 (↑ **0.30**)	81.49 (↓ **1.19**)	55.55 (↓ 0.52)
	Scaffold	62.91 (↑ 1.38)	94.42 (↓ 0.90)	60.70 (↓ **1.01**)
	Scaffold generic	59.07 (↑ **4.02**)	**96.14** (↑ 0.13)	57.68 (↑ 4.02)
	MMP	**50.01** (↓ 11.89)	85.48 (↓ 6.07)	**43.14** (↓ 14.95)
	Similarity (≥0.5)	44.61 (↓ 7.22)	78.03 (↓ 4.27)	35.78 (↓ 8.75)
MMP	Similarity ([0.5,0.7))	**41.88** (↓ **4.87**)	**65.57** (↓ **2.52**)	**27.94** (↓ **5.02**)
only	Similarity (≥0.7)	45.48 (↓ **19.61**)	68.53 (↓ **14.15**)	33.78 (↓ **22.29**)
	Scaffold	44.83 (↓ 16.70)	87.47 (↓ 7.85)	40.86 (↓ 18.83)
	Scaffold generic	42.21 (↓ 12.84)	**88.81** (↓ 7.20)	38.60 (↓ 15.06)
	MMP	**56.43** (↓ 5.47)	86.93 (↓ 4.68)	**50.51** (↓ 7.59)
	Similarity (≥0.5)	49.57 (↓ 2.26)	81.49 (↓ 0.81)	42.09 (↓ 2.44)
Similarity (≥0.5)	Similarity ([0.5,0.7))	**45.75** (↓ **1.00**)	**67.98** (↓ **0.11**)	**32.09** (↓ **0.87**)
only	Similarity (≥0.7)	55.08 (↓ **10.01**)	78.87 (↓ 3.81)	45.24 (↓ **10.83**)
	Scaffold	52.94 (↓ 8.59)	88.48 (↓ **6.84**)	48.70 (↓ 7.99)
	Scaffold generic	49.75 (↓ 5.30)	**90.11** (↓ 5.90)	46.06 (↓ 7.60)
	MMP	**49.65** (↓ 12.25)	87.77 (↓ 3.78)	44.84 (↓ 13.25)
	Similarity (≥0.5)	46.21 (↓ 5.62)	77.17 (↓ 5.13)	36.94 (↓ 7.59)
Scaffold generic	Similarity ([0.5,0.7))	**43.38** (↓ **3.37**)	**64.78** (↓ 3.31)	**28.88** (↓ **4.08**)
only	Similarity (≥0.7)	47.53 (↓ **17.56**)	74.83 (↓ **7.85**)	37.33 (↓ **18.74**)
	Scaffold	48.86 (↓ 12.67)	94.85 (↓ 0.47)	47.19 (↓ 9.50)
	Scaffold generic	47.07 (↓ 7.98)	**96.26** (↑ **0.25**)	**45.89** (↓ 7.77)
	MMP	**58.60** (↓ 3.3)	88.75 (↓ 2.80)	**53.51** (↓ 4.58)
	Similarity (≥0.5)	51.29 (↓ 0.54)	81.76 (↓ 0.54)	43.77 (↓ 0.76)
Merged	Similarity ([0.5,0.7))	**47.16** (↑ **0.41**)	**67.88** (↓ **0.21**)	**33.01** (↑ **0.05**)
	Similarity (≥0.7)	57.48 (↓ **7.61**)	79.23 (↓ 3.45)	47.50 (↓ **8.57**)
	Scaffold	55.54 (↓ 5.99)	91.33 (↓ **3.99**)	52.25 (↓ 7.44)
	Scaffold generic	52.43 (↓ 2.62)	**92.95** (↓ 3.06)	49.84 (↓ 3.82)

Performance on test sets where large property changes are desired

Table S5: Performance comparison of Transformer models trained on different types of molecular pairs on different test sets where big property changes are desired (numbers in bracket represent the absolute increase/decrease compared to the corresponding Transformer model performance on the original test set in Table 4). The extremes (best/worst performance or largest/smallest change) are highlighted in bold.

Test set	Type of molecular pair where Transformer is trained	Successful property constraints (%)	Successful structure constraints (%)	Successful property and structure constraints (%)
MMP	MMP	**44.61** (↓ 17.29)	86.54 (↓ 5.01)	**40.65** (↓ 17.44)
	Similarity (≥0.5)	40.96 (↓ 10.87)	74.32 (↓ 7.98)	31.36 (↓ 13.17)
	Similarity ([0.5,0.7))	39.48 (↓ **7.27**)	**65.17** (↓ **2.92**)	**26.22** (↓ **6.74**)
	Similarity (≥0.7)	38.64 (↓ **26.45**)	67.77 (↓ **14.91**)	27.45 (↓ **28.62**)
	Scaffold	**36.99** (↓ 24.54)	87.44 (↓ 7.88)	33.19 (↓ 26.5)
	Scaffold generic	38.20 (↓ 16.85)	**89.14** (↓ 6.87)	35.01 (↓ 18.65)
Similarity (≥0.5)	MMP	**41.88** (↓ 20.02)	84.17 (↓ 7.38)	**37.19** (↓ 20.9)
	Similarity (≥0.5)	40.83 (↓ 11)	75.56 (↓ 6.74)	31.84 (↓ 12.69)
	Similarity ([0.5,0.7))	39.35 (↓ **7.4**)	**66.02** (↓ **2.07**)	26.91 (↓ **6.05**)
	Similarity (≥0.7)	37.46 (↓ **27.63**)	68.99 (↓ **13.69**)	26.71 (↓ **29.36**)
	Scaffold	**37.07** (↓ 24.46)	88.42 (↓ 6.9)	33.90 (↓ 25.79)
	Scaffold generic	38.26 (↓ 16.79)	**90.63**(↓ 5.38)	35.57 (↓ 18.09)
Similarity ([0.5,0.7))	MMP	**41.27** (↓ 20.63)	84.33 (↓ 7.22)	**36.58** (↓ 21.51)
	Similarity (≥0.5)	40.27 (↓ 11.56)	75.04 (↓ 7.26)	31.11 (↓ 13.42)
	Similarity ([0.5,0.7))	39.37 (↓ **7.38**)	**66.03** (↓ **2.06**)	27.05 (↓ **5.91**)
	Similarity (≥0.7)	**36.07** (↓ **29.02**)	67.95 (↓ **14.73**)	**24.97** (↓ **31.1**)
	Scaffold	36.33 (↓ 25.2)	88.66 (↓ 6.66)	33.36 (↓ 26.33)
	Scaffold generic	37.71 (↓ 17.34)	**90.89** (↓ 5.12)	35.11 (↓ 18.55)
Similarity (≥0.7)	MMP	**45.07** (↓ 16.83)	82.97 (↓ 8.58)	**40.42** (↓ 17.67)
	Similarity (≥0.5)	41.94 (↓ 9.89)	77.54 (↓ 4.76)	33.84 (↓ 10.69)
	Similarity ([0.5,0.7))	40.21 (↓ **6.54**)	**65.76** (↓ **2.33**)	**27.06** (↓ **5.9**)
	Similarity (≥0.7)	43.62 (↓ **21.47**)	72.96 (↓ **9.72**)	34.06 (↓ 22.01)
	Scaffold	**40.14** (↓ 21.39)	86.97 (↓ 8.35)	36.20 (↓ **23.49**)
	Scaffold generic	40.41 (↓ 14.64)	**89.60** (↓ 6.41)	37.34 (↓ 16.32)
Scaffold	MMP	40.00 (↓ 21.9)	82.95 (↓ 8.6)	35.69 (↓ 22.4)
	Similarity (≥0.5)	39.47 (↓ 12.36)	76.50 (↓ 5.8)	31.35 (↓ 13.18)
	Similarity ([0.5,0.7))	38.00 (↓ **8.75**)	**65.47** (↓ 2.62)	**25.59** (↓ **7.37**)
	Similarity (≥0.7)	**37.38** (↓ **27.71**)	68.37 (↓ **14.31**)	27.42 (↓ **28.65**)
	Scaffold	**42.17** (↓ 19.36)	94.00 (↓ 1.32)	**40.21** (↓ 19.48)
	Scaffold generic	41.08 (↓ 13.97)	**95.29** (↓ **0.72**)	39.62 (↓ 14.04)
Scaffold generic	MMP	39.44 (↓ 22.46)	83.57 (↓ 7.98)	35.05 (↓ 23.04)
	Similarity (≥0.5)	39.41 (↓ 12.42)	74.94 (↓ 7.36)	30.47 (↓ 14.06)
	Similarity ([0.5,0.7))	38.09 (↓ **8.66**)	**64.65** (↓ 3.44)	**25.39** (↓ **7.57**)
	Similarity (≥0.7)	**36.69** (↓ **28.4**)	69.13 (↓ **13.55**)	26.66 (↓ **29.41**)
	Scaffold	39.22 (↓ 22.31)	92.53 (↓ 2.79)	37.11 (↓ 22.58)
	Scaffold generic	**40.01** (↓ 15.04)	**94.45** (↓ **1.56**)	**38.45** (↓ 15.21)
Merged	MMP	**40.82** (↓ 21.08)	83.89 (↓ 7.66)	**36.12** (↓ 21.97)
	Similarity (≥0.5)	39.81 (↓ 12.02)	75.00 (↓ 7.30)	30.70 (↓ 13.83)
	Similarity ([0.5,0.7))	38.33 (↓ **8.42**)	**66.64** (↓ **1.45**)	25.94 (↓ **7.02**)
	Similarity (≥0.7)	**36.14** (↓ **28.95**)	68.57 (↓ **14.11**)	**25.58** (↓ **30.49**)
	Scaffold	36.50 (↓ 25.03)	89.17 (↓ 6.15)	33.60 (↓ 23.09)
	Scaffold generic	37.78 (↓ 17.27)	**91.30** (↓ 4.71)	35.26 (↓ 18.40)
